# The relationship between expression of PD-L1 and HIF-1α in glioma cells under hypoxia

**DOI:** 10.1186/s13045-021-01102-5

**Published:** 2021-06-12

**Authors:** Xing-chen Ding, Liang-liang Wang, Xue-dong Zhang, Jun-long Xu, Pei-feng Li, Hua Liang, Xian-bin Zhang, Li Xie, Zi-han Zhou, Jia Yang, Ralph R. Weichselbaum, Jin-ming Yu, Man Hu

**Affiliations:** 1 Department of Radiation Oncology, Shandong Cancer Hospital and Institute, Shandong First Medical University and Shandong Academy of Medical Sciences, No. 440 Ji Yan Road, Jinan, Shandong China; 2 Department of Radiation and Cellular Oncology, Ludwig Center for Metastasis Research, The University of Chicago, Chicago, IL 60637 USA; 3 Department of Pathology, Liaocheng Hospital of China, Liaocheng, Shandong China; 4 Department of Pathology, The 960Th Hospital of PLA, Jinan, Shandong China; 5 Department of General Surgery, Shenzhen University General Hospital & Carson International Cancer Research Centre, Shenzhen, China; 6 Department of Oncology, Weifang Medical University, Weifang, Shandong China

**Keywords:** PD-L1, HIF-1 α, Glioma, Hypoxia, Immunotherapy

## Abstract

Hypoxia inducible factor-1α (HIF-1α) up-regulates the expression of programmed death ligand-1 (PD-L1) in some extracranial malignancies. However, whether it could increase PD-L1 expression in intracranial tumor is still unknown. Here, we explored the relationship between HIF-1α and PD-L1 expression in glioma, and investigated their clinical significance. In glioma patients, HIF-1α and PD-L1 were overexpressed in high grade glioma tissues and were significantly associated with poor survival. In glioma cells, PD-L1 expression was induced under hypoxia condition, and the enhanced PD-L1 expression was abrogated by either HIF-1α knock-down or HIF-1α inhibitor treatment. Furthermore, ChIP-qPCR analysis showed the direct binding of HIF-1α to PD-L1 proximal promoter region, providing evidence that HIF-1α up-regulates PD-L1 in glioma. In glioma murine model, the combination treatment with HIF-1α inhibitor and anti-PD-L1 antibody caused a more pronounced suppressive effect on tumor growth compared to either monotherapy. Immunologically, the combination treatment improved both dendritic cell (DC) and CD8^+^ T cell activation. Overall, our results demonstrated that positive correlation between PD-L1 and HIF-1α in glioma, and provide an alternative strategy, inhibiting HIF-1α, as combination therapies with immunotherapies to advance glioma treatment.

**Supplementary Information:**

The online version contains supplementary material available at 10.1186/s13045-021-01102-5.

**To the Editor**

In clinical, the blockade of the PD-1/PD-L1 pathway havn’t been well-confirmed to prolong OS of glioma patients [1, 2]. With increasing malignancy, hypoxia as a major tumor microenvironment factor widely exhibit in glioma [3], however, the influence of hypoxia on tumor immune escape remains unclearly. Here, we aimed to explore the relationship between the PD-L1 and HIF-1α in glioma, and to investigate their prognostic values.

PD-L1 is wildly used as a candidate biomarker for predicting patients that would respond to anti-PD-1/PD-L1 immunotherapy [4, 5], but not in glioma [6]. We analyzed RNA-seq data from a cohort (640 glioma patients) in CGGA dataset and found that PD-L1 is positively correlated with HIF-1α (Additional file 1: Fig. S1, Additional file 2: S2). To determine this, the PD-L1 and HIF-1α levels in 120 glioma patients’ tissues were detected by immunohistochemical (Fig. 1a, Additional file 4: Table S1). Fifty patients (41.7%) were classified as PD-L1 positive (≥ 5%). PD-L1 was positively associated with tumor grade (Fig. 1b, Additional file 5: Table S2). Moreover, our clinical data showed that high PD-L1 was significantly related to high HIF-1α (*r* = 0.412, *P* < 0.001) (Fig. 1b). These findings were consistent with the external central nervous system tumors [7, 8].

**Fig. 1:**
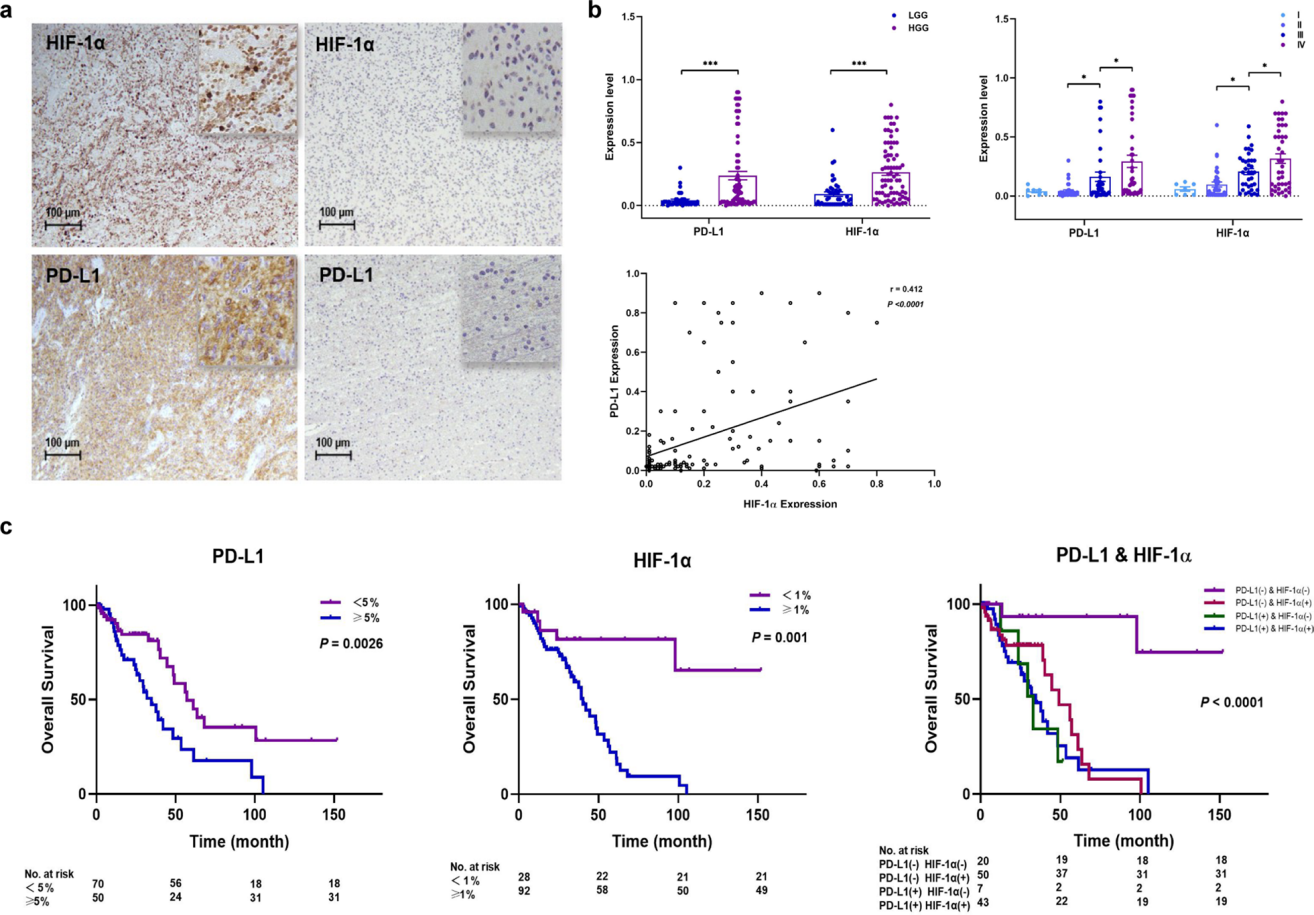
The relationship of PD-L1 and HIF-1α expression in tumor tissue of glioma patients and their impact on the overall survival. **a** Immunohistochemistry (IHC) analysis of HIF-1α and PD-L1 in tissue sections of glioma patients. Typical image of positive expressions of HIF-1α (≥ 1%) and PD-L1(≥ 5%) in tissue sections of one patient with grade IV glioma; Typical image of negative expressions of HIF-1α (< 1%) and PD-L1 (< 5%) in tissue sections of one patient with grade II glioma. **b** PD-L1 and HIF-1α expression in patients with different grades glioma**.** PD-L1 and HIF-1α expressions in high-grade glioma (HGG) group and low-grade glioma (LGG) group; PD-L1 and HIF-1α expressions in grade II to grade III groups; Correlation analysis of PD-L1 and HIF-1α expression (*r* = 0.412, *P* < 0.001) in all glioma patients in our cohort. For (A) to (B), the data were presented as mean ± SEM. *P < 0.05, ***P < 0.001. **c** The overall survival of glioma patients. Statistical significance was determined by log-rank (Mantel-Cox) test

Next, we investigated the correlation of PD-L1/HIF-1α expression and the OS of these glioma patients. The OS in either PD-L1 or HIF-1α positive group was significantly poorer than that in negative group (Fig. 1c). Subsequently, we classified all the patients with combining PD-L1 and HIF-1α expression into four subgroups. The Kaplan–Meier curves indicated that the patients in PD-L1(+) HIF-1α (+) group had worse OS than those in PD-L1(−) HIF-1α (−) group (*P* < 0.0001) (Fig. 1c). Univariate analysis identified PD-L1 ≥ 5%, HIF-1α ≥ 1%, HGG, and older age as unfavorable prognostic predictors (Additional file 6: Table S3). Multivariate analysis was also performed and indicated that both PD-L1 and HIF-1α expression were independent poor prognostic factors (Additional file 6: Table S3). We also found the consistent results in primary glioma patients in CGGA dataset (Additional file 2: Fig. S2).

To further verify the relationship between PD-L1 and HIF-1α, we cultured U251 and U343 glioma cell lines under hypoxic condition for different time and detected the PD-L1 expression. The western blot results showed higher HIF-1α and PD-L1 levels in hypoxia condition (1% O_2_) for either 24-, 48-, or 72-h culturing than those in control condition (21% O_2_) (Additional file 3: Fig. S3). Given these data, we used 1% O_2_ (72 h) as the hypoxia condition for further experiments. Similarly, we observed that PD-L1 expression was also increased with the hypoxia mimic CoCl_2_ treatment (Additional file 3: Fig. S3).

To further dissect the roles of the HIF-1α in PD-L1 up-regulation under hypoxia, we first knocked down HIF-1α using siRNA or inhibited HIF-1α activity using HIF-1α inhibitor (PX-478) and then detected PD-L1 expression. The results showed that either HIF-1α knockdown or PX-478 treatment can significantly decrease PD-L1 expression in glioma cells under hypoxia (Fig. 2a, b, Additional file 7: Table S4 and Additional file 8: Table S5). Given that HIF-1α protein can activate its target genes via directly binding to their promoter [8, 9], we verified whether PD-L1 is a direct target of HIF-1α in glioma cells using ChIP-qPCR assay. The results showed that HIF-1α directly interacts with the PD-L1 promoter region (~ 0.5 kb proximal to the transcription start site) (Fig. 2c and Additional file 9: Table S6). Furthermore, the co-staining PD-L1 and HIF-1α in glioma murine model showed that PD-L1 was highly express in hypoxic regions of tumors (Fig. 2d). These suggest that hypoxia upregulated PD-L1 via increasing HIF-1α in glioma cells.

**Fig. 2:**
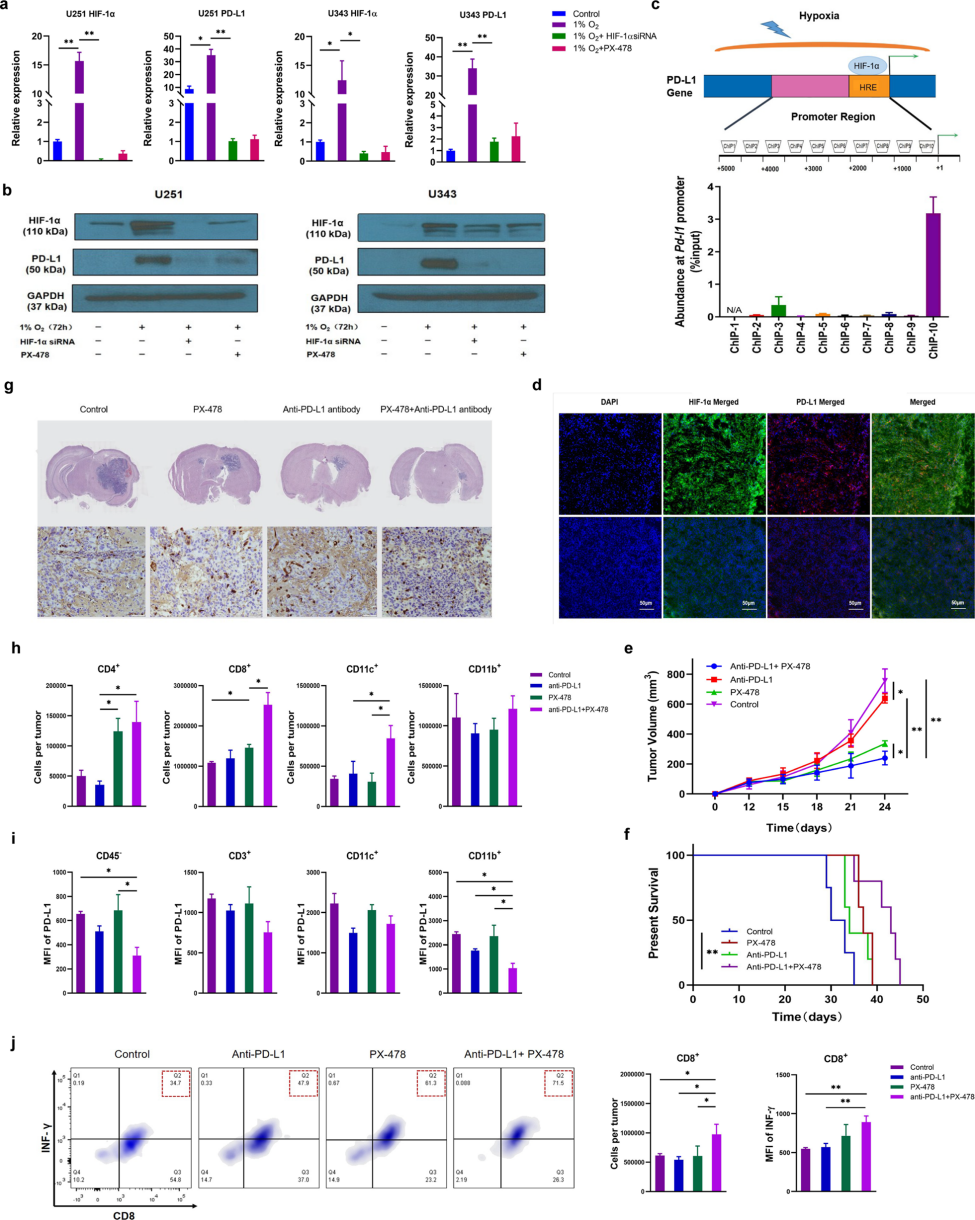
Hypoxia up-regulate PD-L1 expression via HIF-1α in glioma cell lines and combination treatment with HIF-1α inhibitor and anti–PD-L1 antibody can reduce tumor growth in murine model of glioma. **a** qPCR analysis of HIF-1α and PD-L1 mRNA expression in U251 and U343 lines with different treatments as indicated. The qPCR data were normalized to *GAPDH*. The data were presented as mean ± SEM. *P* values were calculated by unpaired two-tailed Student’s t tests. *P < 0.05, **P < 0.01. **b** Western blot analysis of U251 and U343 cells with different treatments using indicated antibodies. **c** Chromatin immunoprecipitation (ChIP) analysis of the PD-L1 promoter in U251 cells using anti-HIF-1α mAb. The experiments were performed in triplicates and repeated three times. **d** Immunofluorescence staining of HIF-1α and PD-L1 expression in tumor cells analyzed by confocal microscopy. Representative images are shown. Scale bars, 50 μm. **e** Mice bearing GL261 cells were divided into the indicated treatment groups. The tumor volumes of mice treated with control, anti–PD-L1 monoclonal antibody, HIF-1α inhibitor (PX-478), or combined anti–PD-L1 antibody and PX-478 were measured and plotted (*n* = 5). Tumor volume was measured twice weekly. Data are presented as mean ± SEM. and the statistical significance was determined by two-way ANOVA**. f** Survival from mice receiving the indicated treatments as described in **e**. Statistical significance was determined by log-rank (Mantel-Cox) test. For (e) to (f) *P < 0.05, **P < 0.01. **g** The HE staining of intracranial tumor and immunohistochemistry analysis of CD8^+^ T cells in intracranial tumor from mice receiving control, anti–PD-L1 antibody, PX-478, or anti–PD-L1 antibody and PX-478. **h** Representative flow cytometry analysis and quantification of CD4^+^ T, CD8^+^ T, CD11c^+^ DC and CD11b^+^ myeloid cells populations in GL261 tumors with the indicated treatments (*n* = 5). **i** Quantification flow cytometry analysis of the PD-L1 expression on CD45^−^, CD3^+^, CD11c^+^ and CD11b^+^ cells (*n* = 5). **j** Representative flow cytometry analysis and quantification of CD8^+^ INF- γ^+^ T cells in GL261 tumors and the MFI of INF- γ in CD8^+^ T cells in U261 tumors at day 14 after treatment (*n* = 5). For (**h**) to (**j**), data are presented as means ± SEM. *P* values were calculated by unpaired two-tailed Student’s t tests. *P < 0.05, **P < 0.01

We hypothesized that combining anti–PD-L1 and HIF-1α inhibitor would trigger an antitumor effect. Thus, we inoculated GL261 cells into wild type mice and treated the mice with anti–PD-L1 antibody and/or HIF-1α inhibitor. The combination treatment exerts a more pronounced antitumor effect, assessed in terms of both tumor growth and survival, than each monotherapy (Fig. 2e, f). Of interest was that, in situ glioma model (Luci^+^GL261), PX-478 can also enhance the intracranial efficacy of anti-PD-L1 antibody (Fig. 2g). Immunologically, our FACS results showed that the combination treatment significantly increased the percentage of tumor-infiltrated CD4^+ ^T, CD8^+ ^T, CD11c^+^ DC (Fig. 2h) and also decreased PD-L1 expression (Fig. 2i). Moreover, we also found the increased numbers of cytotoxic CD8^+ ^T cells (IFNγ^+^CD8^+^) (Fig. 2j). Collectively, these indicate that combination treatment can reverse the immunosuppression microenvironment in glioma.

Our study demonstrated that positive relationship between HIF-1α and PD-L1 in glioma and provide the evidence that targeting HIF-1α can boost anti-PD-1/PD-L1 efficacy for glioma treatment.

## Supplementary Information

**Additional file 1: Fig. S1**. The relationship of PD-L1 and HIF-1α mRNA expression in glioma samples from the Chinese Glioma Genome Atlas (CGGA) dataset. **a.** The relationship of PD-L1expression and different clinical factors, including grade, IDH-1mutant, MGMT methylated status and 1p19q deletion status. **b.** The relationship of HIF-1α expression and different clinical factors, including grade, IDH-1mutant, MGMT methylated status and 1p19q deletion status. **c.** Correlation analysis of PD-L1 and HIF-1α expression in glioma patients. **d.** Correlation analysis of PD-L1, HIF-1α expression and different clinical factors in glioma patients.

**Additional file 2: Fig. S2**. The impact of PD-L1 and HIF-1α mRNA expression on the overall survival (OS) in primary or recurrent glioma patients in CGGA dataset. a-b. the OS of patients with primary or recurrent glioma in CGGA dataset that was stratified by high versus low PD-L1 (a) or HIF-1α level (b). **c.** Correlation analysis of PD-L1 and HIF-1α expression in primary and recurrent glioma patients.

**Additional file 3: Fig. S3**. Western blot analysis and quantification of PD-L1 and HIF-1α expression in glioma cell lines. **a**. Western blot analysis of U251 cell line under 21% O_2_ (72 h), 1% O_2_ (24 h), 1% O_2_ (48 h), 1% O_2_ (72 h) and hypoxia mimic CoCl_2_ (24 h), respectively using anti-PD-L1 antibody and HIF-1α inhibitor. **b**. Quantification analysis of PD-L1 and HIF-1α expression in U251 cells (as a). **c**. Western blot analysis of U343 cell line with indicated treatments using anti-PD-L1 and anti-HIF-1α antibodies. **d**. Quantification analysis of PD-L1 and HIF-1α expression in U343 cells (as c). The data were presented as mean ± SEM. *P < 0.05, **P < 0.01, ***P < 0.001.

**Additional file 4: Table S1**. Patient and tumor characteristics.

**Additional file 5: Table S2**. The expression of PD-L1 and HIF-1α in different grades of glioma patients.

**Additional file 6: Table S3**. Univariate and multivariate Cox regression of glioma patients.

**Additional file 7: Table S4**. Primers used in qRT-PCR.

**Additional file 8: Table S5**. siRNA Target Sequences.

**Additional file 9: Table S6.** Primers used in ChIP-qPCR.

## Data Availability

All supporting data are included in the manuscript and supplemental files. Additional data are available upon reasonable request to corresponding authors.

## Abbreviations

HIF-1α: Hypoxia inducible factor-1α
PD-L1: Programmed death ligand-1
PD-1: Programmed death-1
LGG: Low grade glioma
HGG: High grade glioma
OS: Overall survival
IHC: Immunohistochemical
CGGA: Chinese Glioma Genome Atlas
ChIP-qPCR: Chromatin immunoprecipitation coupled with quantitative PCR
DC: Dendritic cell
FACS: Fluorescence activated Cell Sorting
