# Supplementary figures and images for: The relationship between expression of PD-L1 and HIF-1α in glioma cells under hypoxia
Source: J Hematol Oncol. 2021 Jun 12;14:92. doi: 10.1186/s13045-021-01102-5 (PMC8199387; doi:10.1186/s13045-021-01102-5)

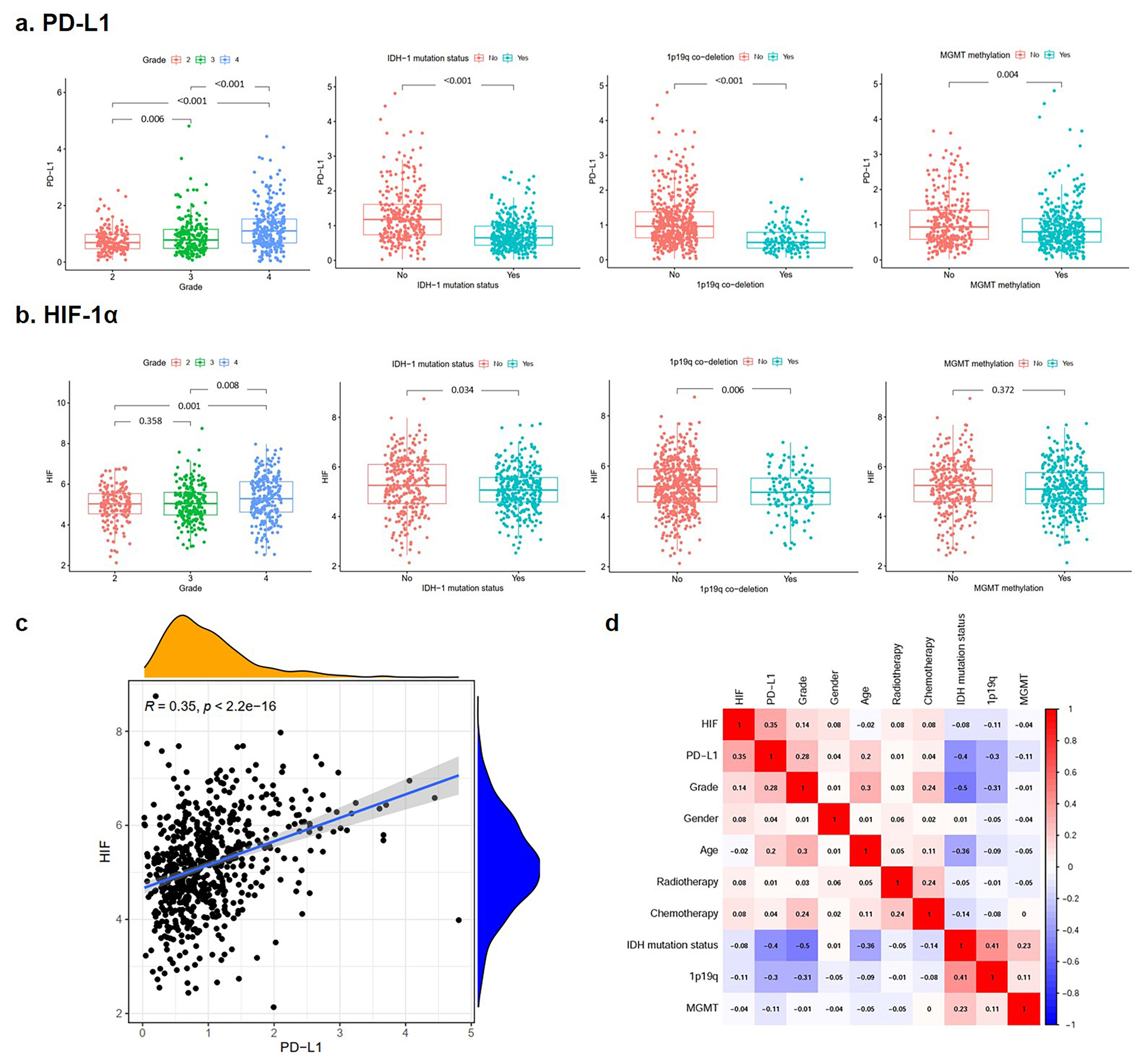

Supplement: Supplementary file 1 — Additional file 1: Fig. S1. The relationship of PD-L1 and HIF-1α mRNA expression in glioma samples from the Chinese Glioma Genome Atlas (CGGA) dataset. a. The relationship of PD-L1expression and different clinical factors, including grade, IDH-1mutant, MGMT methylated status and 1p19q deletion status. b. The relationship of HIF-1α expression and different clinical factors, including grade, IDH-1mutant, MGMT methylated status and 1p19q deletion status. c. Correlation analysis of PD-L1 and HIF-1α expression in glioma patients. d. Correlation analysis of PD-L1, HIF-1α expression and different clinical factors in glioma patients. [file 13045_2021_1102_MOESM1_ESM.jpg]

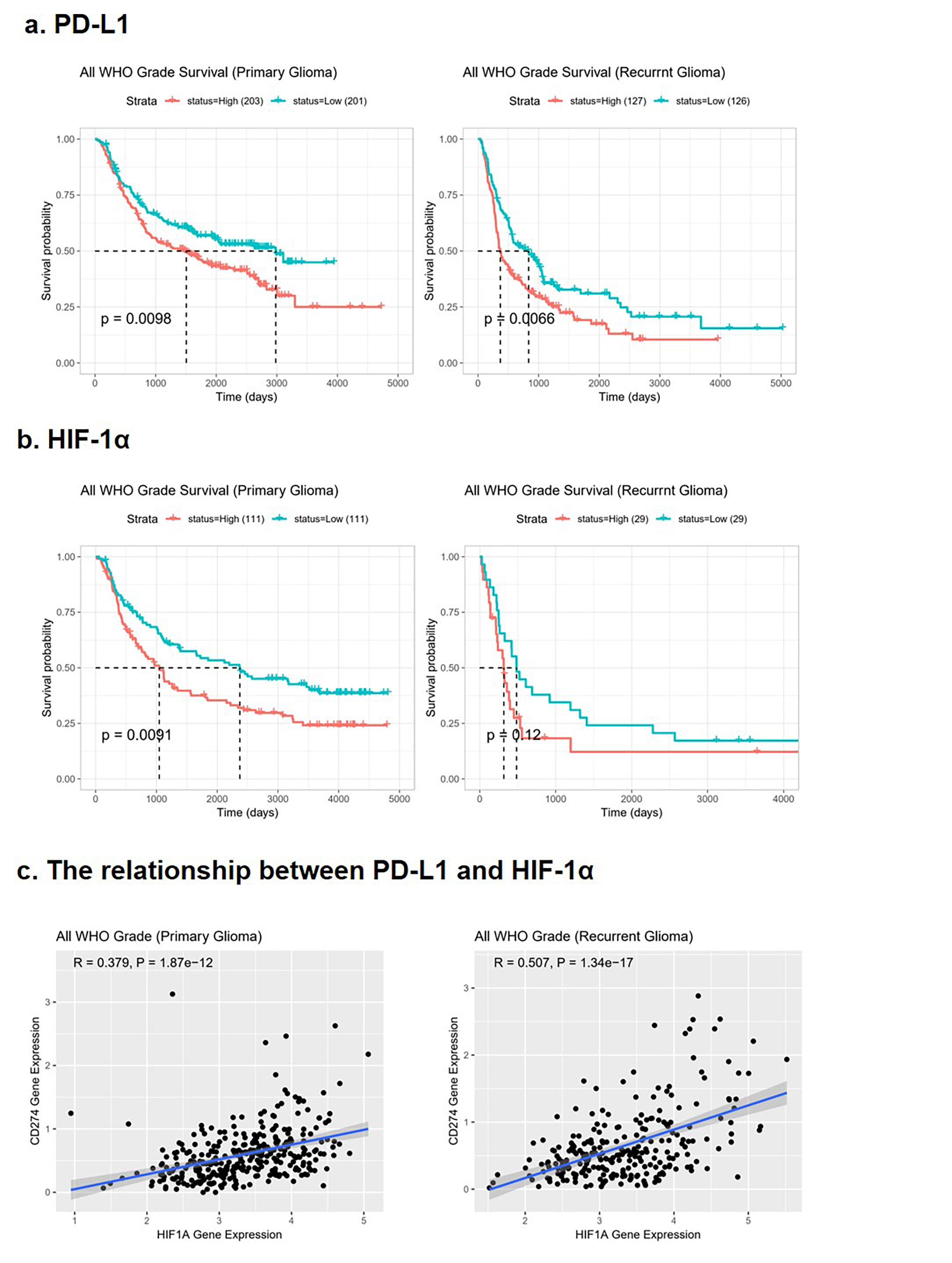

Supplement: Supplementary file 2 — Additional file 2: Fig. S2. The impact of PD-L1 and HIF-1α mRNA expression on the overall survival (OS) in primary or recurrent glioma patients in CGGA dataset. a-b. the OS of patients with primary or recurrent glioma in CGGA dataset that was stratified by high versus low PD-L1 (a) or HIF-1α level (b). c. Correlation analysis of PD-L1 and HIF-1α expression in primary and recurrent glioma patients. [file 13045_2021_1102_MOESM2_ESM.jpg]

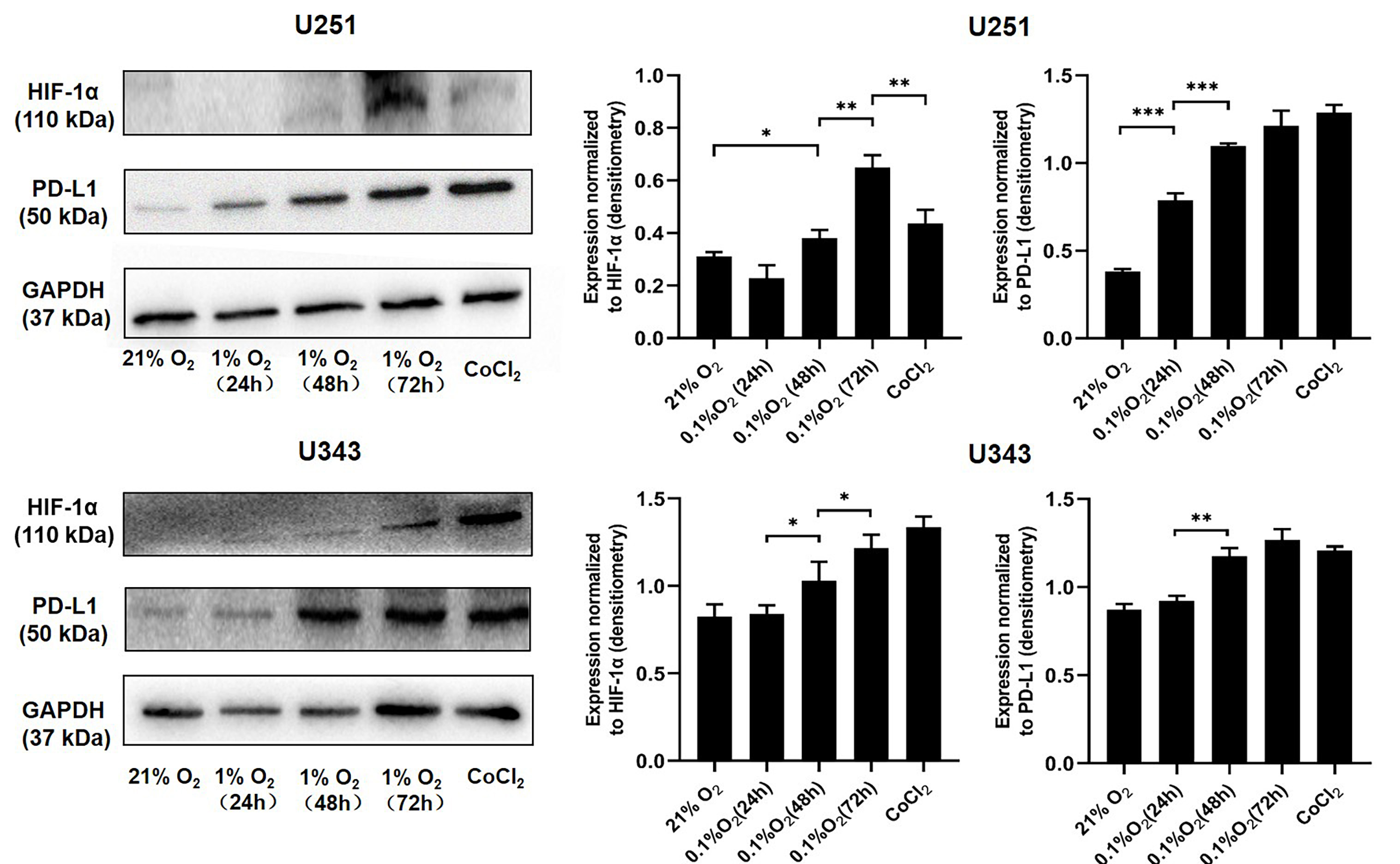

Supplement: Supplementary file 3 — Additional file 3: Fig. S3. Western blot analysis and quantification of PD-L1 and HIF-1α expression in glioma cell lines. a. Western blot analysis of U251 cell line under 21% O2 (72 h), 1% O2 (24 h), 1% O2 (48 h), 1% O2 (72 h) and hypoxia mimic CoCl2 (24 h), respectively using anti-PD-L1 antibody and HIF-1α inhibitor. b. Quantification analysis of PD-L1 and HIF-1α expression in U251 cells (as a). c. Western blot analysis of U343 cell line with indicated treatments using anti-PD-L1 and anti-HIF-1α antibodies. d. Quantification analysis of PD-L1 and HIF-1α expression in U343 cells (as c). The data were presented as mean ± SEM. *P < 0.05, **P < 0.01, ***P < 0.001. [file 13045_2021_1102_MOESM3_ESM.jpg]
