# Supplementary material for: The relationship between expression of PD-L1 and HIF-1α in glioma cells under hypoxia
Source: J Hematol Oncol. 2021 Jun 12;14:92. doi: 10.1186/s13045-021-01102-5 (PMC8199387; doi:10.1186/s13045-021-01102-5)
Supplement: Supplementary file 4 — Additional file 4: Table S1. Patient and tumor characteristics. [file 13045_2021_1102_MOESM4_ESM.docx]

Table S1. Patient and tumor characteristics

| Variate | classify | Number | % |
| --- | --- | --- | --- |
| Age (median age 50) |  |  |  |
|  | ≤ 50 | 59 | 44.2 |
|  | ﹥50 | 61 |  |
| Sex |  |  |  |
|  | Female | 60 | 50 |
|  | Male | 60 | 50 |
| Pathology (WHO) |  |  |  |
|  | I | 7 | 5.8 |
|  | II | 36 | 30.0 |
|  | III | 36 | 30.0 |
|  | IV | 41 | 34.2 |

WHO means World Health Organization
