# Supplementary material for: The relationship between expression of PD-L1 and HIF-1α in glioma cells under hypoxia
Source: J Hematol Oncol. 2021 Jun 12;14:92. doi: 10.1186/s13045-021-01102-5 (PMC8199387; doi:10.1186/s13045-021-01102-5)
Supplement: Supplementary file 5 — Additional file 5: Table S2. The expression of PD-L1 and HIF-1α in different grades of glioma patients. [file 13045_2021_1102_MOESM5_ESM.docx]

Table S2. The expression of PD-L1 and HIF-1α in different grades of glioma patients

|  | PD-L1 |  |  | HIF-1α |  |  |
| --- | --- | --- | --- | --- | --- | --- |
|  | Mean Expression | *P* | *P** | Mean Expression | *P* | *P** |
| LGG (43) | 23.93% ± 28.92% |  | ＜0.001 | 8.7% ± 11.91% |  | ＜0.001 |
| I (7) | 4.09%± 3.25% | 0.979 |  | 5.74% ± 4.87% | 0.653 |  |
| II (36) | 4.33%± 5.76% | 0.017 |  | 9.28%± 12.81% | 0.014 |  |
| HGG (77) | 42.9% ± 5.40 % |  |  | 26.24% ± 22.44% |  |  |
| III (36) | 17.49%± 23.42% | 0.024 |  | 20.49% ± 15.62% | 0.014 |  |
| IV (41) | 29.58%± 32.24% |  |  | 31.30%± 26.21% |  |  |

*P* means the difference of grade I and II, II and III, III and IV respectively.

*P** means the difference of expression between LGG group and HGG group.
