# Supplementary material for: The relationship between expression of PD-L1 and HIF-1α in glioma cells under hypoxia
Source: J Hematol Oncol. 2021 Jun 12;14:92. doi: 10.1186/s13045-021-01102-5 (PMC8199387; doi:10.1186/s13045-021-01102-5)
Supplement: Supplementary file 6 — Additional file 6: Table S3. Univariate and multivariate Cox regression of glioma patients. [file 13045_2021_1102_MOESM6_ESM.docx]

Table S3. Univariate and multivariate Cox regression of glioma patients

| Variate | Univariate analysis | | | Multivariate analysis | | |
| --- | --- | --- | --- | --- | --- | --- |
| OS | *P* value | HR | 95% CI | *P* value | HR | 95% CI |
| Age  (≤50 or >50) | 0.024^*^ | 1.925 | 1.091 – 3.396 | 0.087 | 1.661 | 0.930 – 2.969 |
| Sex  (Female or male) | 0.649 | 1.141 | 0.646 - 2.014 |  |  |  |
| Grade  (LGG or HGG) | 0.005^*^ | 2.565 | 1.324 - 4.971 | 0.661 | 0.809 | 0.314 – 2.085 |
| PD-L1 Expression  (＜5% or ≥ 5%) | 0.001^*^ | 2.551 | 1.432 – 4.546 | 0.028^*^ | 1.943 | 1.075 – 3.511 |
| HIF-1α Expression  (＜1% or ≥ 1%) | 0.002^*^ | 3.758 | 1.645 – 8.583 | 0.021^*^ | 2.763 | 1.167 – 6.543 |

*P* means the difference between different groups; HR means Hazard Ratio; CI means Confidence Interval; * means *P*< 0.05.
