# Supplementary material for: The relationship between expression of PD-L1 and HIF-1α in glioma cells under hypoxia
Source: J Hematol Oncol. 2021 Jun 12;14:92. doi: 10.1186/s13045-021-01102-5 (PMC8199387; doi:10.1186/s13045-021-01102-5)
Supplement: Supplementary file 7 — Additional file 7: Table S4. Primers used in qRT-PCR. [file 13045_2021_1102_MOESM7_ESM.docx]

| **Primers used in qRT - PCR** | | |
| --- | --- | --- |
| Name | Sequence | |
| PD-L1 | F | 5’- GCTGCACTAATTGTCTATTGGGA -3’ |
|  | R | 5’- AATTCGCTTGTAGTCGGCACC -3’ |
| HIF-1α | F | 5’- TCCTTCGGACACATAAGCTCC -3’ |
|  | R | 5’- GACAGAAAGATCATGTCACCGT -3’ |
| GAPDH | F | 5’- TGCACCACCAACTGCTTAGC -3’ |
|  | R | 5’- GGCATGGACTGTGGTCATGAG -3’ |

Table S4：Primers used in qRT - PCR
