# Supplementary material for: The relationship between expression of PD-L1 and HIF-1α in glioma cells under hypoxia
Source: J Hematol Oncol. 2021 Jun 12;14:92. doi: 10.1186/s13045-021-01102-5 (PMC8199387; doi:10.1186/s13045-021-01102-5)
Supplement: Supplementary file 8 — Additional file 8: Table S5. siRNA Target Sequences. [file 13045_2021_1102_MOESM8_ESM.docx]

| **siRNA Target Sequences** | | |
| --- | --- | --- |
| siRNA | Sequence | |
| HIF-1α | sense | 5’- CUGAUGACCAGCAACUUGA - 3’ |
|  | antisense | 5’- UCAAGUUGCUGGUCAUCAG - 3’ |
| Control siRNA | sense | 5’- CGUACGCGGAAUACUUCGA - 3’ |
|  | antisense | 5’- UCGAAGUAUUCCGCGUACG - 3’ |

Table S5：siRNA Target Sequences
