# Supplementary material for: The relationship between expression of PD-L1 and HIF-1α in glioma cells under hypoxia
Source: J Hematol Oncol. 2021 Jun 12;14:92. doi: 10.1186/s13045-021-01102-5 (PMC8199387; doi:10.1186/s13045-021-01102-5)
Supplement: Supplementary file 9 — Additional file 9: Table S6. Primers used in ChIP-qPCR. [file 13045_2021_1102_MOESM9_ESM.docx]

| **Primers used in ChIP-qPCR** | | |
| --- | --- | --- |
| Name | Sequence | |
| hPDL1_chip_1 | F | 5’-TGTACTTACCTTCGAGTCTCT- 3’ |
|  | R | 5’-CTGAGGCTTGCTATTAACCA- 3’ |
| hPDL1_chip_2 | F | 5’-GTGTGAGTATGTATCTTCCTTG- 3’ |
|  | R | 5’-AGTTGCCTGATGAATGTTCT- 3’ |
| hPDL1_chip_3 | F | 5’-AGTCCTCAAGGCTCTTCA- 3’ |
|  | R | 5’-TTAGTTATGGTGGTCAGGAA- 3’ |
| hPDL1_chip_4 | F | 5’-TTGTTGGAGGTCTCATCTT- 3’ |
|  | R | 5’-CTCATCTGACACTCACAATC- 3’ |
| hPDL1_chip_4 | F | 5’-GGAAACAGAGGAAGAGAAATG- 3’ |
|  | R | 5’-AGTGGACCTGAAGAGATGT- 3’ |
| hPDL1_chip_6 | F | 5’-GAAGGAAGGATGGTACTGATA- 3’ |
|  | R | 5’-GGTCTTGGAGGTCAACATT- 3’ |
| hPDL1_chip_7 | F | 5’-ACACGAATCCTCACATTACT- 3’ |
|  | R | 5’-AATCATATCCTCCTAGATGGC- 3’ |
| hPDL1_chip_8 | F | 5’-TTCGGGAACTTTGGGAAG- 3’ |
|  | R | 5’-GCTGACACTGCCTTGATT- 3’ |
| hPDL1_chip_9 | F | 5’-ATTATGACACCATCGTCTGT- 3’ |
|  | R | 5’-TCGTGGATTCTGTGACTTC- 3’ |
| hPDL1_chip_10 | F | 5’-CAGATGTTGGCTTGTTGTAA- 3’ |
|  | R | 5’-GTATCTAGTGTTGGTGTCCTA- 3’ |

Table S6: Primers used in ChIP-qPCR
